# Advances in Intraperitoneal Chemotherapy for Gastric Cancer Patients with Peritoneal Metastases: Current Status of Treatment and Institutional Insights

**DOI:** 10.3390/jcm14103521

**Published:** 2025-05-17

**Authors:** Shin Saito, Hironori Yamaguchi, Akira Saito, Yuki Kaneko, Hideyuki Ohzawa, Shinichiro Yokota, Joji Kitayama

**Affiliations:** 1 Department of Surgery, Jichi Medical University, Saitama 330-8503, Japan; yamaguchi@jichi.ac.jp (H.Y.); saito.akira@jichi.ac.jp (A.S.); lotte.yk@gmail.com (Y.K.); froyokota@hotmail.com (S.Y.); kitayama@jichi.ac.jp (J.K.); 2 Division of Clinical Oncology, Jichi Medical University, Saitama 330-8503, Japan; 92015ho@jichi.ac.jp; 3 Division of Abdominal Transplant, Department of Surgery, Stanford University School of Medicine, Stanford, CA 94305, USA

**Keywords:** intraperitoneal chemotherapy, paclitaxel, gastric cancer with peritoneal disease, conversion surgery

## Abstract

**Introduction:** Peritoneal metastasis (PM) is the most common site of recurrence following curative resection for advanced gastric cancer (GC). Along with disease progression, it can lead to complications such as intestinal obstruction, hydronephrosis, obstructive jaundice, and ascites, significantly impairing the patient’s quality of life. Therefore, peritoneal metastasis is considered a critical target for treatment. In general, these patients are treated with systemic chemotherapy; however, the therapeutic effect is often limited due to the anticancer agents’ poor penetration into the peritoneal cavity. We aim to identify factors associated with the best overall survival (OS) in GC patients with peritoneal metastasis. **Methods:** Patients with advanced GC who were diagnosed as having macroscopic PM or positive peritoneal cytology by staging laparoscopy were enrolled. We introduced intraperitoneal Paclitaxel (IP-PTX) combined with S-1 plus oxaliplatin (SOX). Gastrectomy with lymph node dissection was performed as conversion surgery when the PM showed an excellent response. **Results:** Ninety-six patients received IP-PTX + SOX, with a median of 16 courses. The 1- and 5-year OS rates were 70.2% and 24.5%, respectively, with a mean survival time (MST) of 20.0 months. No chemotherapy-related mortality was observed. Conversion surgery was performed in 44 patients (45.8%), with a 1-year OS rate of 100%. **Conclusions:** Combination chemotherapy using the IP-PTX + SOX regimen is highly effective and is recommended as induction chemotherapy for patients with PM from GC. Conversion gastrectomy should be considered following an excellent response, particularly in patients with peritoneal cancer index (PCI) scores below 20.

## 1. Introduction

Although the incidence of gastric cancer (GC) has declined over the past half-century, it still accounts for 4.9% of new malignant tumors, ranking fifth in both incidence and mortality, and remains a significant global concern [1]. The prognosis of patients with gastric cancer with peritoneal metastasis (PM) remains poor, highlighting the urgent need for new treatment strategies [2,3,4]. PMs are the most common form of metastases and the leading site of recurrence in advanced GC [5,6], making them a critical factor in addressing the disease’s poor prognosis.

Although gastrectomy is not recommended for GC with PM, and systemic chemotherapy is typically used for cases with distant metastasis, the outcome of GC with PM remains unsatisfactory [2]. However, recent advancements in chemotherapy have enabled the use of conversion surgery for stage IV GC in patients who show a favorable response [7].

High prevalence rates of GC continue to be reported in China, South Korea, and Japan, while there are regional differences in the incidence of GC between East Asia and Western countries [8]. Yoo et al. analyzed 2328 patients with GC and found that the most common site of initial recurrence after the curative resection of advanced GC was PM (43.9%), followed by local recurrence (32.5%), and liver metastasis (16.9%) [9]. Furthermore, the overall survival (OS) for GC patients with PM is estimated to be 3–6 months when no treatment is provided [10]. Thus, dissemination is a key factor in the prognosis of GC. PM is considered a critical target for treatment, as it is the most frequent form of metastasis in both simultaneous and recurrent cases of GC [11]. However, PM derived from GC has traditionally been regarded as a terminal condition due to its aggressive nature and has often been inadequately addressed in treatment.

The early identification of PM is critical for improving the outcome of advanced GC. However, detecting PM can be challenging, especially when only a small amount of ascites is present in imaging [12]. Since conventional imaging modalities cannot accurately identify PM, studies have shown that the detection rate improves when diagnostic or staging laparoscopy with cytological washing is performed [13]. Even in clinical trials targeting inoperable recurrent gastric cancer, PM is often excluded due to the difficulty of identifying it in the early stages and accurately assessing it as a target lesion. As a result, there has been limited progress in research.

Despite recent advances in chemotherapy, the prognosis for GC patients with PM remains poor, and standard treatment has yet to be established [2]. Systemic chemotherapy with 5-fluorouracil (5-FU) + cisplatin was proposed as the standard treatment for unresectable or recurrent GC [14], but patients with large amounts of ascites or extensive dissemination were excluded from the study, and its effectiveness in these cases has not been evaluated [7].

The ToGA trial was a pivotal clinical trial that demonstrated a significant overall survival (OS) improvement in patients with HER2-positive unresectable or recurrent GC who received trastuzumab in combination with standard chemotherapy (cisplatin and 5-FU) [15]. However, since PM derived from GC, particularly of the diffuse type, typically exhibits low HER2 expression [16], the therapeutic effect of trastuzumab is unlikely to be effective in treating PM.

The JCOG9912 [17] and SPIRITS trials [14] conducted in Japan demonstrated that oral S-1 was non-inferior to 5-FU in terms of OS. However, oral S-1 alone was found to be inferior to cisplatin and S1 combination therapy in patients with HER2-negative unresectable or recurrent GC. Patients with PM often face challenges with oral S-1 administration due to issues such as ascites or intestinal strictures, even though S-1 remains a key chemotherapy option for GC in Asia.

The CheckMate 649 trial demonstrated improved OS and progression-free survival (PFS) with the combination of Nivolumab and conventional chemotherapy for HER2-negative unresectable gastric cancer [18]. However, PM accounted for only 24% of the metastatic sites in the enrolled patients. In the ATTRACTION-4 trial, which involved Japan, South Korea, and Taiwan, PFS was significantly improved with Nivolumab combination chemotherapy for unresectable GC, but no improvement in OS was observed. Additionally, a subgroup analysis of patients with PM showed that Nivolumab did not improve either OS or PFS [19]. Currently, the introduction of Nivolumab has not led to a breakthrough in the treatment of PM in GC.

Peritonectomy and hyperthermic intraperitoneal chemotherapy (HIPEC) have been developed based on the concept that PM can be a localized lesion within the peritoneal cavity [20]. In Western countries, this intensive strategy has proven effective against peritoneal dissemination from conditions such as pseudomyxoma peritonei, mesothelioma, and ovarian cancer [2]. However, the relatively low incidence of GC in Western countries has resulted in limited reports of peritonectomy and HIPEC for the peritoneal dissemination of GC. In Japan, no large-scale randomized control trials (RCTs) have been conducted due to concerns over the high toxicity and surgical complication rates of these treatments [2]. Despite the exploration of various approaches to treat PM, including systemic chemotherapy, targeted molecular chemotherapy, immunotherapy, hyperthermia, and cytoreductive surgery, none have resulted in satisfactory clinical outcomes [21].

In Japan, 5-FU + leucovorin is commonly used for GC patients who are unable to maintain oral intake due to PM or abdominal distention associated with ascites [22]. An RCT comparing 5-FU + leucovorin + paclitaxel (FLTAX) with 5FU + leucovorin for GC patients with severe ascites or PM demonstrated that FLTAX was superior in terms of PFS (median 5.4 months vs. 1.9 months). However, no significant difference was observed in OS (Median 7.3 months vs. 6.1 months) [22].

The recent SPOTLIGHT and GLOW trials have demonstrated that Zolbetuximab, which is a monoclonal immunoglobulin antibody that targets and binds claudin 18 isoform 2 (CLDN18.2), has been shown to improve the outcome of patients with unresectable metastatic GC, particularly with undifferentiated histology [23,24]. However, a previous study reported that CLDN18.2 is rarely expressed in peritoneal lesions in many patients, suggesting that targeting CLDN18.2 with Zolbetuximab might be less effective in some patients with peritoneal involvement [25].

Fluoropyrimidine- and platinum-based chemotherapy has remained the first-line treatment for HER2-negative, advanced gastric cancer over the past decade [26]. Currently, patients with PM are typically treated with systemic chemotherapy, similarly to patients with metastases at other sites. However, it is widely recognized that the effectiveness of systemic chemotherapy alone is limited for treating peritoneal lesions [27].

Intraperitoneal chemotherapy serves as a localized treatment targeting diseases within the peritoneal cavity and should not be directly compared to systemic chemotherapy. The intraperitoneal administration of chemotherapy allows for extremely high concentrations of the drugs to directly reach the cancer lesions within the peritoneal cavity [21]. In fact, this method increases both the exposure time and concentration of chemotherapy at peritoneal lesions, while reducing systemic toxicity [2].

The Phoenix-GC trial, which compared intraperitoneal and intravenous paclitaxel plus S-1 versus cisplatin plus S-1, demonstrated that the mean survival time (MST) of the intraperitoneal chemotherapy group was 17.7 months, though the MST of the latter group was 15.2 months. This trial did not demonstrate statistical superiority for intraperitoneal paclitaxel combined with systemic chemotherapy [28]. The authors in this trial noted that the results were significantly affected by the baseline imbalance between the groups, particularly regarding the number of ascites, which may have favored the cisplatin plus S-1 arm. They concluded that the exploratory analyses suggested potential clinical benefits of intraperitoneal paclitaxel (IP-PTX) for GC [28]. A randomized control trial (the Phoenix-GC2 trial) is currently underway to compare intraperitoneal combination chemotherapy with systemic chemotherapy in patients with scirrhous type GC with the goal of preventing PM.

Intraperitoneal chemotherapy using cisplatin and PTX has shown favorable outcomes for ovarian cancer with PM in the United States [29]. Due to its lipid-soluble properties, PTX forms a polymeric structure in aqueous solutions and is slowly absorbed through the lymphatic system, allowing for the maintenance of extremely high concentrations of the drug in the peritoneal cavity for an extended period of time [30].

When PTX is administered intraperitoneally, it is expected to exhibit significant antitumor activity against peritoneal deposits while minimizing systemic toxicity [27].

Here, we describe an overview of chemotherapy for GC with PM and the role of conversion surgery following a favorable response to chemotherapy, based on our experiences.

We tried to identify factors associated with the best OS in GC patients with PM.

## 2. Material and Methods

### 2.1. Patients and Treatment

Since 2016, we have introduced IP-PTX combined with S-1 plus oxaliplatin (SOX) for GC with PM. Below is an overview of our institution’s treatment experience.

Patients diagnosed with advanced GC underwent staging laparoscopy and were enrolled into a treatment group when macroscopic disseminated metastases (P1) or positive peritoneal cytology (CY1) were confirmed. The eligibility and exclusion criteria have been reported previously [27].

The eligibility criteria included histologically proven unresectable adenocarcinoma, peritoneal dissemination diagnosed by staging laparoscopy, good performance status, adequate bone marrow function (leukocyte count, 3000–12,000/mm^3^), hemoglobin > 8.0 g/dL and platelet count >100,000/mm^3^, and adequate liver and renal function.

Exclusion criteria included distant metastasis other than peritoneal or ovarian metastasis, and previous palliative gastrectomy. Written informed consent was obtained from all patients. This study was carried out in accordance with the Declaration of Helsinki of 1975 and was approved by the Institutional Review Board of Jichi Medical University.

**Figure 1:**
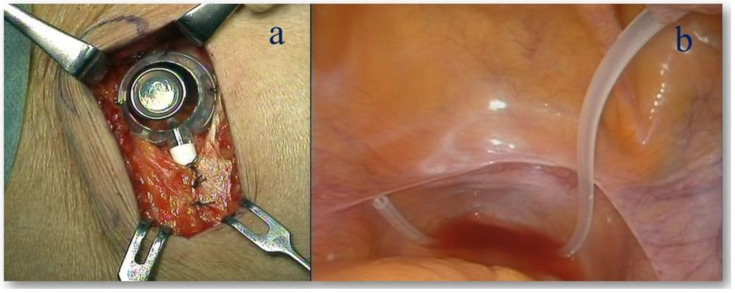
Intraperitoneal access port. The IP port is subcutaneously implanted in the right lower quadrant of the abdomen (**a**) and the tip of the catheter is placed in the pelvic space (**b**).

During staging laparoscopy, an access port is placed subcutaneously, and the tip of the catheter is directed into the pelvis and positioned in the Douglas fossa (Figure 1).

Intraperitoneal access ports not only facilitate the administration of anticancer drugs but also enable the collection of ascites fluid, which allows for cytological examination over time. The extent of peritoneal dissemination is assessed using the Peritoneal Cancer Index (PCI) score [31]. PTX is administered intraperitoneally at 40 mg/m^2^ on days 1 and 8. Oxaliplatin was given intravenously at 100 mg/m^2^ on day 1, and S-1 was administered orally at 80 mg/m^2^/day for 14 consecutive days, with cycles repeated every 21 days. When the macroscopic regression of peritoneal lesions is observed, along with negative peritoneal cytology and the absence of other distant metastases, gastrectomy with lymph node dissection is performed as conversion surgery. After the gastrectomy, the same regimen continues as long as possible.

### 2.2. Assessment of Response and Toxicity

Tumor responses were evaluated after several (up to ten) courses of treatment and categorized based on the RECIST guidelines (version 1.1). The volumes of malignant ascites and peritoneal cytology were also considered to assess antitumor effects.

Toxicity was graded according to the National Cancer Institute’s Common Terminology Criteria for Adverse Events, version 4.0.

### 2.3. Statistical Analysis

The overall survival (OS) rates were estimated according to the Kaplan–Meier method. OSl curves were compared using the log-rank test, and a *p* value < 0.05 was considered to be statistically significant.

We also tried to determine which factors correlated with good OS.

## 3. Results

### 3.1. Intraperitoneal Administration of Paclitaxel (IP-PTX) Combined with S-1 Plus Oxaliplatin (SOX) for GC with PM

**Figure 2:**
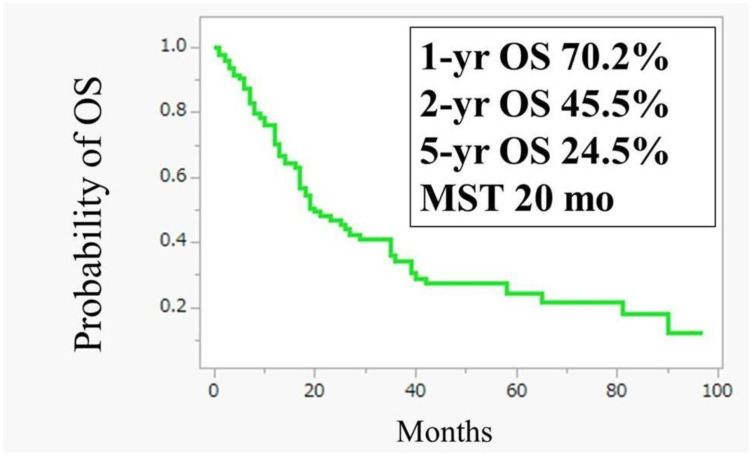
Kaplan–Meier analysis of overall survival (OS) of 96 patients with peritoneal metastasis.

As of May 2024, 96 patients received IP-PTX combined with SOX, with a median of 16 courses (range: 1–97). Oxaliplatin was discontinued after a median of 6.5 courses due to the hematotoxicity or intolerable peripheral neuropathy. Malignant ascites resolved or decreased in 40 out of 55 patients (73%), and peritoneal cytology converted to negative in 46 out of 65 patients (70%). The 1- and 5-year OS rates were 70.2% and 24.5%, respectively, with an MST of 20.0 months (Figure 2).

In total, 96 GC patients with PM were enrolled in this study for IP-PTX +SOX. The 1- and 5-year overall survival (OS) rates were 70.2% and 24.5%, respectively, with a median survival time (MST) of 20.0 months.

The most frequently observed grade 3/4 toxicities were neutropenia (30.2%), anemia (4.3%), and anorexia (4.3%). Complications associated with the peritoneal access device included intraperitoneal catheter obstruction in five patients (5.2%) and access port infection in four patients (4.2%). All these patients required surgical intervention. There was no treatment-related mortality.

### 3.2. Conversion Surgery

**Figure 3:**
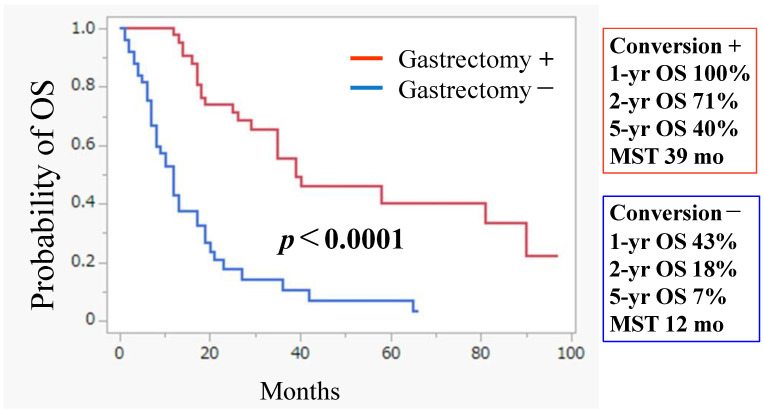
Kaplan–Meier plot analysis of overall survival (OS) of patients who did (*n* = 44) and did not (*n* = 52) undergo conversion gastrectomy.

Conversion surgery was performed in 44 patients (45.8%) who demonstrated a macroscopic reduction in PM with 1- and 5-year OS rates of 100% and 40%, respectively. Their MST was 39.0 months (Figure 3). Histological examination revealed grade 2 and grade 3 histological responses in ten (22.7%) and three (6.8%) patients, respectively. In contrast, the 1- and 5-year OS rates for patients who did not undergo conversion gastrectomy were 43.0 and 7.0% with an MST of 12.0 months—significantly lower than those who underwent gastrectomy (*p* < 0.0001) (Figure 3).

The 1- and 5-year OS rates of the patients with excellent response to IP-PTX + SOX who underwent gastrectomy showed 1- and 5-year OS rates of 100% and 40%, respectively. Their MST was 39.0 months. The 1- and 5-year OS rates of the patients who did not undergo conversion gastrectomy were 43.0 and 7.0%, respectively, with an MST of 12.0 months, which was significantly lower than those in patients who underwent gastrectomy (*p* < 0.0001).

**Figure 4:**
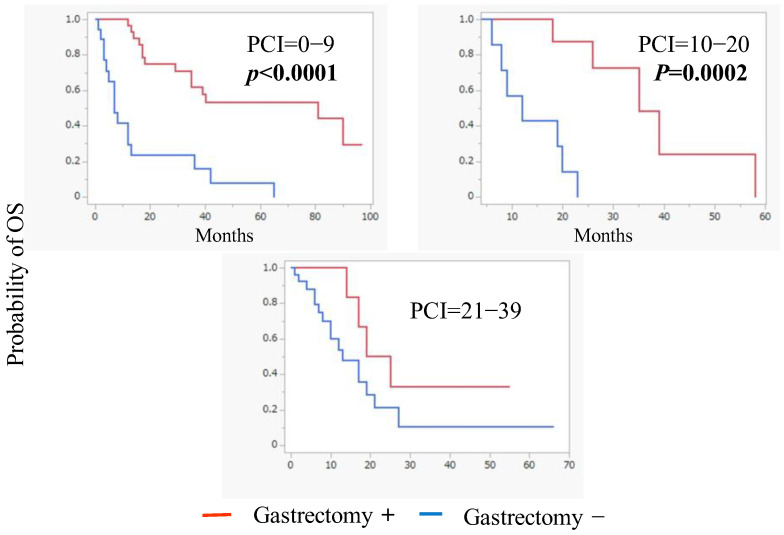
Kaplan–Meier plot analysis of overall survival (OS) of patients who did and did not undergo conversion gastrectomy according to peritoneal cancer index (PCI) scores of 1–9 (*n* = 41), 10–20 (*n* = 20), and 20–39 (*n* = 35).

Based on the PCI scores, a subgroup analysis revealed that conversion surgery was associated with improved OS in patients with PCI scores below 20 points (Figure 4).

Subgroup analysis showed that conversion surgery could significantly improve the OS in patients with PCI scores of less than 20 points.

Of the 44 patients who underwent conversion surgery, 25 showed recurrence, with the most frequent site being peritoneal disease in 20 patients.

## 4. Discussion

### 4.1. Promising Strategy Against Peritoneal Disease

Currently, a standard treatment strategy for GM with PM has yet to be established, although recent advancements in chemotherapy, including immune checkpoint inhibitors and monoclonal antibodies targeting CLDN18.2, have shown significant progress.

For decades, PM from GC has been considered a terminal condition, and treatment has basically been palliative.

The repeated intraperitoneal infusion of anticancer drugs with taxanes enables the delivery of a high concentration of drugs to the tumor cells in the peritoneal cavity [2]. GC patients with PM treated by a combination chemotherapy protocol, including S1, systemic PTX, and IP-PTX showed better outcomes compared to those treated by systemic chemotherapy alone [28].

The efficacy of systemic chemotherapy for PM is generally limited by the peritoneum–plasma barrier, which restricts the efficient delivery of therapeutic agents from the systemic circulation into the peritoneal cavity [32,33]. In contrast, IP chemotherapy can bypass this barrier and has demonstrated some efficacy against PM originating from GC [28].

Our results (Figure 3) suggest that the curative resection of the primary lesion, following IP chemotherapy for PM, may improve OS in patients with GC and PM. These findings highlight the importance of controlling peritoneal disease to improve prognosis among GC patients with PM. IP chemotherapy—with or without hyperthermia treatment—can play a pivotal role in the management of PM from GC.

IP administration of PTX offers a pharmacokinetic advantage, as its hydrophobic nature and high molecular weight allow it to maintain high concentrations within the peritoneal cavity [27]. We think that the factors that might correlate with good OS were the macroscopic disappearance of PM and the negative conversion of cytology after IP-PTX administration. In those conditions, GC patients with PM may be able to undergo curative surgery.

Taken together, IP-PTX combined with systemic chemotherapy and gastrectomy represents a promising therapeutic strategy for managing GC with PM.

In the ATTRACTION-4 trial, a subgroup analysis showed that the median overall survival for unresectable GC patients with PM was 13.67 months in the nivolumab plus chemotherapy group, compared to 15.77 months in the placebo plus chemotherapy group [19]. In contrast, 96 patients treated with IP-PTX combined with SOX therapy had a more favorable outcome with an MST of 20.0 months (Figure 2).

### 4.2. Future Perspectives on Treatment for Peritoneal Disease

Recent studies have shown that exosomes play an important role in cell-to-cell communication through the transfer of DNA, messenger RNA, and microRNA (miR) [34].

miR-21-5p and miR-223-3p have been reported to promote invasion of gastric cancer cells [35,36], suggesting that gastric cancer with serosal invasion may release exosomes including these oncomiR into the peritoneal cavity and assist the progression of peritoneal tumor. Ohzawa et al. reported that the OS of patients with high miR-21/miR-29b or miR-223/miR-29b ratios were significantly worse than that of patients with low ratios [37].

miR29s have been shown to exert inhibitory effects on multiple oncogenic processes, including cell proliferation, epithelial–mesenchymal transition, angiogenesis, and fibrosis [38,39]. One proposed mechanism by which miR29s suppress PM from GC is through the inhibition of mesothelial mesenchymal transition (MMT) in the peritoneum [40].

The peritoneum consists of a single layer of flat mesothelial cells, and the phenotypic and functional transformation of these mesothelial cells is referred to as mesothelial mesenchymal transition and considered a critical event in the formation of a premetastatic niche that facilitates the implantation and growth of disseminated tumor cells [41,42].

Mesothelial cells can be activated by TGF-β and acquire a mesenchymal phenotype through the MMT, which enhances the migration of disseminated tumor cells to the sub-peritoneal space [43,44,45].

Recent studies described that miR-29 also strongly suppresses the epithelial–mesenchymal transition (EMT) of tumor cells [39,46].

Our previous study demonstrated that IP administration of miR-29b suppressed the development of PM, at least in part, by inhibiting TGF-β1-induced MMT in the peritoneum [47].

Furthermore, miR-29b levels in peritoneal fluids were significantly reduced in patients with PM from gastric cancer, suggesting IP administration of miR-29b could be clinically useful in preventing PM in advanced gastric cancer [47].

Based on these results, we have developed novel therapeutic approaches for PM, including intraperitoneal miR replacement therapy [40]. In murine models, the delivery of miR-29b into the peritoneum using an adeno-associated virus (AAV) vector markedly suppressed PM in both gastric and pancreatic cancer models. Additionally, it enhanced the therapeutic effect of IP-PTX in gastric cancer model [40].

These results highlight gene therapy using tumor suppressor miR as a promising strategy for treating refractory PM.

This study has acknowledged limitations.

There was no assessment regarding the OS of GC patients with PM who continued to receive IP-PTX plus SOX without conversion surgery even though the regimen showed an excellent response for their PM. Because patients with lower PCI scores were more likely to undergo conversion surgery, there might be a selection bias in the Kaplan–Meier plot analysis of OS between patients who underwent gastrectomy and those who did not.

Future studies should evaluate the effect of IP-PTX combination chemotherapy including immune checkpoint inhibitors or zolbetuximab for GC patients with PM.

## 5. Conclusions

Combination chemotherapy using SOX + IP-PTX regimen has demonstrated high efficacy and is now recommended as an induction chemotherapy for patients with PM from GC.

Conversion gastrectomy should be considered following an excellent response, particularly in patients with PCI scores below 20.

The factors that were associated with the best OS rates were those that showed lower PCI scores and miR-21/miR-29b or miR-223/miR-29b ratios in their ascites.

As future directions, intraperitoneal administration of AAV-miR-29b could represent a potential breakthrough in the treatment of PM. Gene therapy using tumor suppressor miR holds promise as a potential strategy for treating refractory PM from GC.

## Data Availability

The datasets used and/or analyzed during the current study are available from the corresponding author upon reasonable request.

## Abbreviations

The following abbreviations are used in this manuscript:

GC	Gastric Cancer
PM	Peritoneal Metastasis
OS	Overall Survival
PFS	Progression Free Survival
HIPEC	Hyperthermic Intraperitoneal Chemotherapy
CLDN18.2	Claudin 18 isoform 2
MST	Mean Survival Time
IP-PTX	Intraperitoneal–Paclitaxel
miR	microRNA
MMT	Mesothelial Mesenchymal Transition
AAV	Adeno-Associated Virus
